# Synergistic Effects between Ambient Air Pollution and Second-Hand Smoke on Inflammatory Skin Diseases in Chinese Adolescents

**DOI:** 10.3390/ijerph191610011

**Published:** 2022-08-13

**Authors:** Mengting Liao, Yi Xiao, Shenxin Li, Juan Su, Ji Li, Bin Zou, Xiang Chen, Minxue Shen

**Affiliations:** 1 Hunan Key Laboratory of Skin Cancer and Psoriasis, Hunan Engineering Research Center of Skin Health and Disease, Department of Dermatology, Xiangya Hospital, Central South University, Changsha 410008, China; 2 National Engineering Research Center of Personalized Diagnostic and Therapeutic Technology, Changsha 410008, China; 3 Health Management Center, Xiangya Hospital, Central South University, Changsha 410008, China; 4 Department of Surveying and Remote Sensing Science, School of Geosciences and Info-Physics, Central South University, Changsha 410008, China; 5 National Clinical Research Center for Geriatric Diseases, Xiangya Hospital, Central South University, Changsha 410008, China; 6 Department of Social Medicine and Health Management, Xiangya School of Public Health, Central South University, Changsha 410008, China

**Keywords:** air pollution, second-hand smoke, synergistic effect, atopic dermatitis, eczema, urticaria, adolescents

## Abstract

Atopic dermatitis (AD), chronic hand eczema (CHE), and urticaria are common inflammatory skin diseases among adolescents and associated with air quality. However, the synergistic effects of ambient air pollution and second-hand smoke (SHS) have been unclear. We conducted a cross-sectional study including 20,138 Chinese college students where dermatological examinations and a questionnaire survey were carried out. A generalized linear mixed model was applied for the association between individualized exposure of O_3_, CO, NO_2_, SO_2_, PM_2.5_, and PM_10_ and the prevalence of inflammatory skin diseases. Interactions between air pollutants and SHS were analyzed. As a result, CO, NO_2_, SO_2_, PM_2.5_, and PM_10_ were positively correlated with the prevalence of AD, CHE, and urticaria. Higher frequency of SHS exposure contributed to increased probabilities of AD (*p* = 0.042), CHE (*p* < 0.001), and urticaria (*p* = 0.002). Of note, CO (OR: 2.57 (1.16–5.69) in third quartile) and NO_2_ (OR: 2.38 (1.07–5.27) in third quartile) had positive interactions with SHS for AD, and PM_2.5_ synergized with SHS for CHE (OR: 2.25 (1.22–4.15) for second quartile). Subgroup analyses agreed with the synergistic results. In conclusion, SHS and ambient air pollution are both associated with inflammatory skin diseases, and they have a synergistic effect on the prevalence of AD and CHE.

## 1. Introduction

Atopic dermatitis (AD), chronic hand eczema (CHE), and urticaria are among the most common inflammatory skin diseases in adolescents. These diseases heavily impair quality of life for patients and impose financial burdens for the society [1,2]. AD and CHE are atopic diseases characterized by intense pruritus and eczematous lesions [3]. Urticaria is pruritic wheal and/or angioedema which can be induced by certain triggering factors [4]. There is a strong link between atopic diseases and urticaria, and they share many similar risk factors [5,6]. It is known that the prevalence rates of atopic disease and skin allergy are higher in more industrialized and developed countries, and those in developing countries are relatively lower [7,8,9]. In addition to genetic factors, environmental determinants contribute to the epidemic variation and partially explain the etiology of atopic diseases.

Accumulating evidence has shown the association between ambient air pollution and atopic skin diseases. NO_2_, SO_2_, CO, or O_3_ were responsible for increased lifetime eczema or diagnosed AD over the last year [10,11,12,13]. PM_2.5_ and PM_10_ were associated with higher prevalence of eczema by some studies but protective factors in others [10,12,14], while some studies demonstrated that air pollution does not necessarily affect the development of eczema or AD [15,16,17]. Disruption of skin barrier integrity, immune-related responses, and alteration of gut microbiota have recently been proposed as the potential mechanisms of the association between air pollutants and atopic skin diseases [18,19,20]. The correlation between urticaria and air pollution has rarely been explored. An air quality health index based on O_3_, NO_2_, and PM2.5 was shown to be positively correlated with emergency department visits for urticaria [18]. Nonetheless, these studies failed to consider individualized metabolism, which influences the actual exposure levels of air pollutants. In addition, previous studies concentrated on younger age groups, mostly preschool children, but prevalence of atopic diseases varied among different ages; however, the effect of air pollution on adolescents is still vague [12,15,16].

Second-hand smoke (SHS) is a behavioral factor that threatens human health, and it is associated with up to 600,000 deaths in nonsmokers each year [21]. In China, smoking is not well controlled [22]. In public places including restaurants, hospitals, and universities, a smoke-free policy has not been implemented. A recent study by our team confirmed that SHS exposure was associated with atopic dermatitis and hand eczema in a dose–response manner, which coincides with a Korean study where SHS incursion into homes was related with allergic symptoms for children [23,24]. However, the relationship of SHS with urticaria was not addressed by previous studies. Furthermore, SHS has been combined with other factors to evaluate the interaction effect on development of allergic diseases. SHS can enhance the adverse health effect of antibiotic use on rhinitis and asthma [25]. However, the interactions between SHS and other ambient air pollutants in atopic diseases are still unclear.

In this study, we conducted a cross-sectional study including 20,138 Chinese college students, to investigate the association of individualized exposure to ambient air pollution with AD, CHE, and urticaria, as well as the interactions between SHS and air pollution. A generalized linear mixed model was applied as an appropriate model to fit the random effect of clusters (city district or county). Importantly, this study fills the gap in large-scale research regarding the prevalence of inflammatory skin diseases and air pollution in Chinese adolescents, as well as adds new knowledge on the synergistic effects between environmental factor and SHS in inflammatory skin diseases.

## 2. Materials and Methods

### 2.1. Study Population

The study was conducted in September and October 2018 on freshman students in five universities at Changsha City, where 20,138 students from all 34 provinces across China were enrolled. The geographical distribution of the students’ origin is shown in Supplementary Figure S1. With the consent to participate, students received an evaluation for dermatologic conditions. The disease diagnosis for each participant was determined by certificated and experienced dermatologists. The diagnostic criteria for AD, CHE, and urticaria included clinical manifestation, disease history, family history, and physical examinations, according to the guidelines from the American Academy of Dermatology [26]. All doctors were trained for the diagnostic criteria before the field survey. More than 80 investigators from different cooperating hospitals participated in the survey. For recurrent symptoms or lesions, only the current manifestation was considered. A questionnaire survey followed immediately, which included demographic information and lifestyle items including activities, sleep and bathing. The detailed questionnaire content relevant to this study is shown in Table S1. This study was approved by the medical ethics committee of Xiangya Hospital, Central South University.

### 2.2. Environmental Factors

Ambient air pollution levels of O_3_, CO, NO_2_, SO_2_, PM_2.5_, and PM_10_, as well as humidity and temperature, were obtained from the 6 year long high-resolution air quality dataset over China from 2013 to 2018 [27]. Of the 20,138 participants, 19,064 were linked to environmental data by city district or county code of the participants’ hometown, whereas the other 1074 had incomplete information of their hometown location. To fully consider individual differences, adjusted daily exposure to pollutants was estimated using the following procedure: (1) calculate the mean concentration of air pollutants for 2013 to 2018; (2) estimate basal metabolic rate (BMR) by individual age and sex according to a nationwide study among the Chinese population [28]; (3) estimate the metabolic equivalents (METs) for school time (assuming 40 h/week for all students), physical activities (self-reported type, frequency, and duration), and sleep (self-reported). The information on individual activities and sleep was obtained from the questionnaire survey, the details of which can be found in Table S1; (4) adjust BMR by METs; (5) estimate the METs-weighted respiratory rate; (6) estimate averagely daily exposure (mg/kg·day or μg/kg·day) to pollutants according to the 6 year mean concentrations and respiratory rate [3,29]. Consequently, the adjusted daily dose of each pollutant for each participant was calculated. Details can be found in our previous publication [30].

### 2.3. Covariates

For covariates, sex and family income were considered as demographic variables; SHS, bath frequency, and BMI were included as behavioral habit variables; humidity and temperature were treated as environmental covariates [21,29]. Information on sex (male/female), age (years), second-hand smoke (SHS) (never, <1 day/week, and ≥1 day/week), family annual income (<10,000, 10,000–29,999, 30,000–49,999, 50,000–99,999, 100,000–199,999, and >200,000 CNY/year), frequency of bathing (≤1/week, 2–4/week, 5–7/week, and ≥8/week) was obtained through the questionnaire survey mentioned above. The body mass index (BMI) was calculated as weight/squared height (kg/m^2^) obtained from physical examinations.

### 2.4. Statistical Analysis

Statistical analyses were performed using SPSS version 23 (IBM SPSS Statistics 23) and R version 4.0.4. Continuous data were presented as the mean ± standard deviation (SD) and compared using ANOVA for differences between groups, whereas categorical data were presented as the number (%) and compared using the chi-square test. Covariates were compared between case and control populations for AD, CHE, and urticaria. The adjusted daily dose of exposure to each pollutant was divided into quartiles according to 25th, 50th, and 75th percentiles to evaluate the odds ratio (OR) for diseases.

The generalized linear mixed model was applied to estimate the association between ambient air pollution and prevalence of AD, CHE, and urticaria, where city (the city from which the student comes) was treated as a random effect. The analysis consisted of unadjusted and adjusted models. The unadjusted model described the crude OR; in the adjusted model, OR was adjusted for demographic variables (sex and family income), behavioral habits (bath frequency and BMI), and environmental factors (humidity and temperature). The model was established using package “lme4” in R. Subgroup analysis was performed on the basis of the adjusted model. A depiction of the linear association between air pollutants and disease prevalence was further performed using package “ggplot2”.

To assess the synergistic effects between air pollutants and SHS for the prevalence of AD, CHE, and urticaria, the generalized linear mixed model was applied, with city as a random effect, and the model was adjusted for demographic, behavioral, and environmental variables.

## 3. Results

The study population included 19,064 college students (51.5% of male) with a mean age of 18.3 years. The general characteristics of the whole population are summarized in Table 1. Among these students, 481 (2.5%), 560 (2.9%), and 1332 (7.0%) were diagnosed with AD, CHE, and urticaria, respectively. The distribution of daily dose of exposure to O_3_, CO, NO_2_, SO_2_, PM_2.5_, and PM_10_ level is summarized for the total population and separately for case and control for AD, CHE, and urticaria in Table 2. The levels of each pollutant were divided into four quartiles to assess OR for diseases.

**Figure 1:**
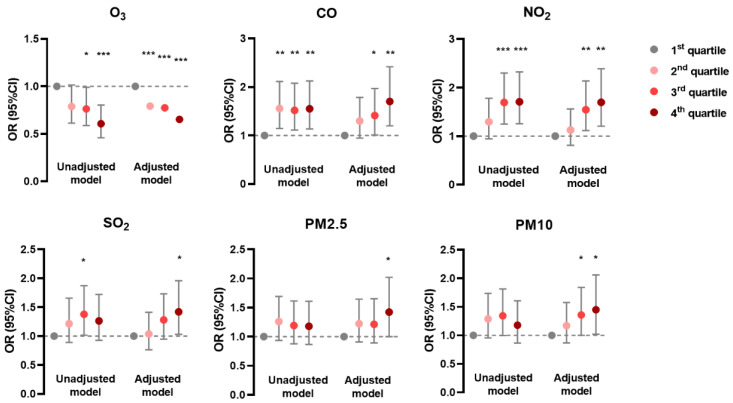
Association between atopic dermatitis and adjusted daily dose of ambient air pollutants. The unadjusted model describes the crude OR; the adjusted model was adjusted for demographic variables, behavioral habits, and environmental factors. * 0.01 < *p* < 0.05; ** 0.001 < *p* < 0.01; *** *p* < 0.001.

**Figure 2:**
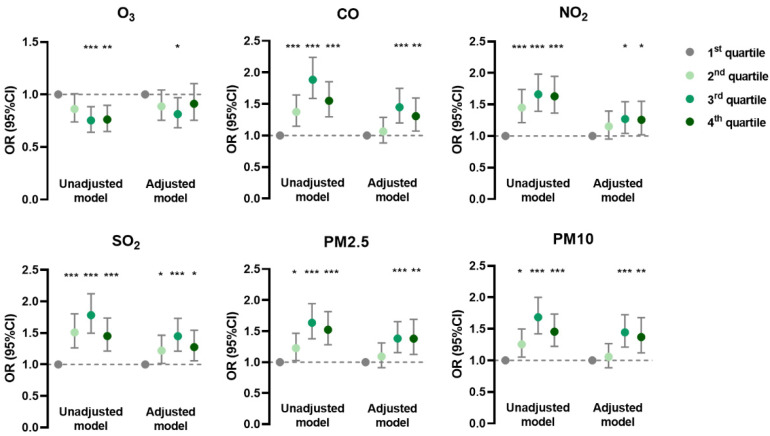
Association between chronic hand eczema and adjusted daily dose of ambient air pollutants. The unadjusted model describes the crude OR; the adjusted model was adjusted for demographic variables, behavioral habits, and environmental factors. * 0.01 < *p* < 0.05; ** 0.001 < *p* < 0.01; *** *p* < 0.001.

**Figure 3:**
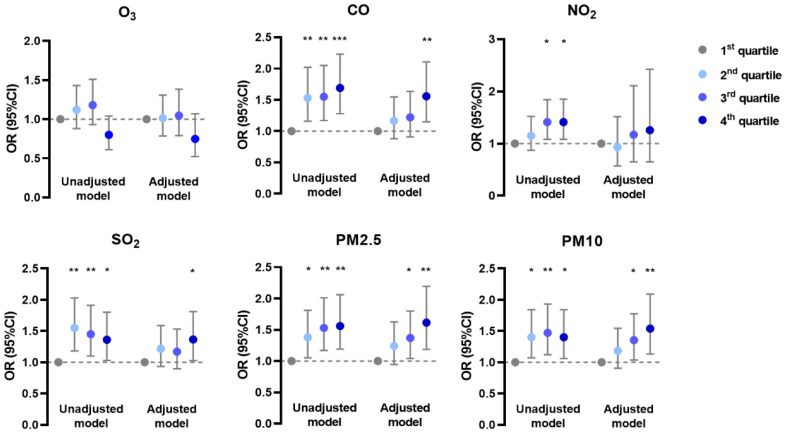
Association between urticaria and adjusted daily dose of ambient air pollutants. The unadjusted model describes the crude OR; the adjusted model was adjusted for demographic variables, behavioral habits, and environmental factors. * 0.01 < *p* < 0.05; ** 0.001 < *p* < 0.01; *** *p* < 0.001.

In the generalized linear mixed model, the prevalence of AD was negatively associated with O_3_ and positively associated with CO, NO_2_, and SO_2_ in unadjusted and adjusted models; it was positively associated with PM_2.5_ and PM_10_ mainly in the adjusted model (Figure 1). The prevalence of CHE had positive correlations with CO, SO_2_, PM_2.5_, and PM_10_ in both models, but with NO_2_ only in the unadjusted model (Figure 2). Meanwhile, the prevalence of urticaria increased with higher levels of CO, NO_2_, SO_2_, PM_2.5_, and PM_10_, but decreased with O_3_ in both models (Figure 3). Linear associations between air pollutants and the prevalence of AD, CHE, and urticaria are shown in Figures S2–S4.

The association of second-hand smoke (SHS) with AD, CHE, and urticaria was analyzed. Notably, a higher frequency of SHS exposure was positively correlated with the prevalence of all three diseases (Table 3). Other covariates including sex, family income, BMI, and bath frequency for AD, CHE, and urticaria are shown in Table S2.

Furthermore, the synergistic effects between SHS and air pollution exposure were estimated. As the proportions of students with SHS exposure for <1 day/week and ≥1 day/week were relatively low, we combined these two groups as the SHS exposure population, which was compared to the group with no SHS exposure. As shown in Table 4, CO had a positive interaction with SHS for AD (OR (95% CI): 2.57 (1.16–5.69) for third quartile, *p* = 0.020), suggesting that students who were exposed to SHS, compared to those without SHS exposure, were more likely to have AD under higher exposure levels of CO. Therefore, SHS enhanced the adverse effects of CO for AD. Similarly, NO_2_ exposure synergized with SHS for AD (OR (95% CI): 2.38 (1.07–5.27) for third quartile, *p* = 0.032). Meanwhile, PM_2.5_ had an interaction with SHS for CHE (OR (95% CI): 2.25 (1.22–4.15) for second quartile, *p* = 0.009). However, NO_2_ had a negative interaction with SHS for urticaria (OR (95% CI): 0.65 (0.44–0.97) for fourth quartile, *p* = 0.034).

**Figure 4:**
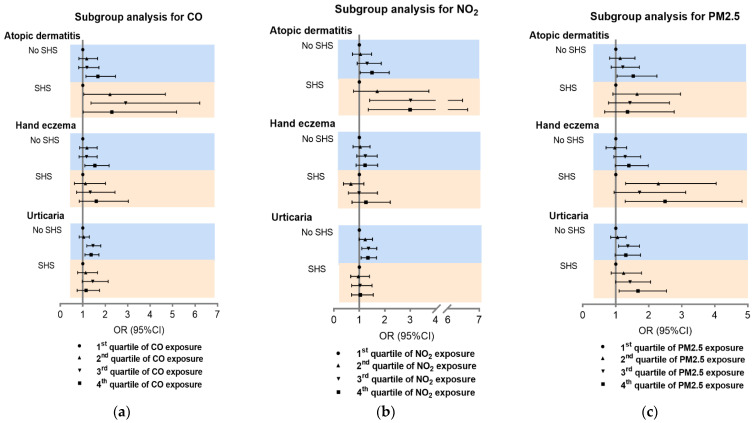
Subgroup analysis of association between CO, NO_2_, and PM_2.5_ and inflammatory skin diseases. Subgroup analysis for CO (**a**), NO_2_ (**b**) and PM_2.5_ (**c**). SHS: second-hand smoke.

On the basis of the synergistic effects above, SHS subgroup analysis was carried out for the association of CO, NO_2_, and PM_2.5_ with inflammatory skin diseases. Consistent with the synergistic analysis results, the effects of CO and NO_2_ were greater on AD in students with SHS exposure than those without SHS (Figure 4a,b). Furthermore, PM_2.5_ had a more significant effect on CHE in students with SHS compared to those without SHS (Figure 4c). Subgroup analysis for O_3_, SO_2_, and PM_10_ showed a lower association of SHS with disease prevalence (Figure S5).

## 4. Discussion

In the present study, we found a significant correlation of AD and urticaria with O_3_, CO, NO_2_, SO_2_, PM_2.5_, and PM_10_, and of CHE with CO, SO_2_, PM_2.5_, and PM_10_. SHS contributed to a higher prevalence of AD, CHE, and urticaria. SHS had synergistic effects with NO_2_ and CO on AD, and with PM_2.5_ on CHE.

This is the first epidemic research showing the negative correlation between O_3_ and AD. A Korean study with elementary school children and a Belarusian study with 0–2 year old kids both showed that O_3_ level contributed to a high risk of AD [11,13]. Another cross-sectional study including over 1,000,000 children in Taiwan found no correlation between O_3_ and AD [17]. To our surprise, we found that O_3_ was negatively associated with AD prevalence. Interestingly, recent studies raised that O_3_ could potentially serve as a therapeutic regimen for AD. In a randomized controlled clinical trial, patients had a significant decrease in SCORAD scores and inflammatory cell infiltration in AD lesions after 3 day ozone therapy (ozone hydrotherapy followed by ozonated oil) [31]. Mechanistically, O_3_ has a bactericidal effect on *Staphylococcus aureus* (*S. aureus*), which accounts for 90% of the microbiome in AD lesions [31]. Another fundamental study revealed that ozonated oil decreased the Th2-dominant cytokines response and increased IL-10 expression, thereby suppressing inflammation in AD murine model [32]. However, whether O_3_ interferes with the original development and protects against the onset of AD is still unknown.

We found that PM_2.5_ and PM_10_ were both positively associated with AD. Particulate matter has been shown to be related with not only respiratory diseases (COPD and asthma) but also lung cancer, mental disorders, and pregnancy outcomes [33,34,35,36,37]. The correlation between particulate matter and atopic dermatitis is inconsistent. A France study showed that a higher level of PM_10_ increased the risk of both lifetime AD and AD in the past year [38]. PM_2.5_ increased the risk of lifetime AD by a Germany study with a population aged over 70 [14], while a US study showed that PM_2.5_ and PM_10_ were negatively associated with AD [10]. Results from our study agreed with the positive association. Research on the mechanism behind this association has been limited. In a mouse model study, particulate matter exposure upregulated the expression of *SPRR2D*, *S100A9*, *STFA3*, *CHIL1*, *DBP*, and *IL1B*, which are responsible for skin barrier integrity and immune response [18]. Another group revealed that skin inflammation induced by ambient particulate is attributable to oxidative stress or programmed cell death [19].

Other pollutants including NO_2_, SO_2_, and CO were all positively associated with AD in our study. The association between NO_2_ and AD was consistent a previous study with over 90,000 individuals aged 0–17 years in the US and a case–control study with 0–2 year old children in Belarus [10,13]. The association of CO with AD was less frequently reported, but our result agrees with the Belarusian study mentioned above [13]. Interestingly, another study from Shanghai, China, showed that SO_2_ was not associated with AD [12]. The difference could derive from the different sample sizes and age groups, as their study was performed on 3358 preschool children, while ours included 19,064 adolescents. In addition, only the fourth quartile of SO_2_ exposure level was significantly correlated with AD in our study, suggesting that the association was not necessarily distinct.

Chronic hand eczema was found to be positively associated higher levels of CO, SO_2_, PM_2.5_, and PM_10_ in our study. Although there is sufficient research on ambient air pollution and AD, studies on hand eczema are rarely reported. A cross-sectional study in southern Sweden showed that living within 100 m of a road with more than 10 cars per minute was associated with hand eczema during the last 12 months; however, the classification of air pollution source was not specified [39]. Therefore, our study adds new knowledge on the correlation between ambient air pollution and chronic hand eczema. It was observed that particulate matter, especially diesel exhaust particles (DEPs), could change the composition and function of the gut microbiota [20]. Furthermore, there was a varied abundance of specific bacterial genera in children who developed IgE-associated allergic disease [40]. This suggests a potential contributing role of particulate matter in the development of allergic diseases via an altered gut microbiota. Moreover, the immune tolerance through the generation of peripheral antigen experienced T_reg_ cells could be related to the mechanism [41,42].

A significant association between CO, SO_2_, PM_2.5_, and PM_10_ and urticaria was demonstrated in our results. This is consistent with the previous finding that positive results were observed between air quality health index (AQHI) and odds ratio for emergency department visits for urticaria in Canada [43]. Although AQHI in their study was based on O_3_, NO_2_, and PM_2.5_, they did not specify the association between each air pollutant and visits for urticaria. Another study from the US showed that the ground-level O_3_ was positively associated with angioedema [44], while our results showed that the third quartile of adjusted O_3_ level was negatively associated with the prevalence of urticaria. The effect of O_3_ on urticaria still needs further exploration.

In this study, we found that SHS was positively correlated with inflammatory skin diseases. Smoking has been identified as a risk factor for AD and CHE, but the association of exposure to SHS with urticaria is barely understood. Our results add more evidence that SHS is hazardous to the health of nonsmoker adolescents. Previous studies showed that SHS induced IL-1β, IL-6, IL-13, and TNF-α secretion from the nasal cavity or saliva, as well as increased CD4^+^ T cells and IgE in the periphery, thus promoting inflammatory processes and immunological responses [45,46,47]. The association between SHS and inflammatory skin diseases could be due to a similar mechanism, but this still needs confirmation.

Furthermore, a positive interaction between SHS and air pollution has been found. SHS is a source of indoor particulate matter, while air pollution is an outdoor environmental factor. Exposure to both indoor and outdoor air pollution might have a synergistic effect. Recently, SHS was shown to break the homeostasis of cholesterol and bile acid metabolism, as well as change the composition of gut microbiota [48]. Combined with the finding that ambient air pollution, especially particulate matter, also changed the composition and function of the gut microbiota, we might speculate that SHS and ambient air pollution synergistically affect the prevalence of skin inflammatory diseases via mechanisms involving gut microbiota [20].

This study had several strengths. First, we used individualized exposure level for all pollutants, with the consideration that differences in age, sex, and lifestyle including activities and sleep influenced the metabolism and the actual exposure level for individuals. Second, instead of inquiring self-reported symptoms or diagnosis recalled by the participants, we evaluated our participants for dermatological conditions by physicians, thus avoiding recall bias. Third, this study found for the first time a positive interaction between ambient air pollution and SHS, providing evidence for better control of air quality and SHS.

This study had some limitations. First, we assumed that participants were exposed to only an outdoor environment and did not take indoor air quality into consideration. However, indoor particular matter and gas pollution levels are influenced by ventilation and outdoor levels; hence, the indoor air quality is not completely independent of outdoor factors [49,50]. Additionally, the actual indoor pollution levels for all participants are not feasible to obtain. Second, no causation can be concluded from the study since it had a cross-sectional design. However, we retrospectively collected data on air pollution during the last 6 years and diagnosed the skin diseases at the timepoint of research. To some extent, this might suggest that the diseases developed after the air pollution exposure.

## 5. Conclusions

In conclusion, ambient air pollution (NO_2_, SO_2_, CO, PM_2.5_, and PM_10_) and second-hand smoke exposure are associated with a higher prevalence of atopic dermatitis, chronic hand eczema, and urticaria among Chinese adolescents. Second-hand smoke interacts with NO_2_ and CO to enhance the adverse effect on atopic dermatitis, and it also synergizes with PM_2.5_ to increase the possibility of chronic hand eczema.

## Figures and Tables

**Table 1:** General information of total study population (*n* = 19,064).

Characteristic	Mean ± SD or *n* (%)
Age (years, mean ± SD)	18.3 ± 0.7
Sex (*n*, %)	
Male	9813 (51.5%)
Female	9251 (48.5%)
Second-hand smoking (*n*, %)	
Never	15,100 (79.2%)
<1 day/week	2597 (13.6%)
≥1 day/week	1367 (7.2%)
Family annual income (CNY, *n*, %)	
<10,000	2046 (10.7%)
10,000–29,999	4151 (21.8%)
30,000–49,999	3316 (17.4%)
50,000–99,999	4211 (22.1%)
100,000–199,999	3825 (20.1%)
≥200,000	1515 (7.9%)
BMI (*n*, %)	
<19.00	3781 (19.8%)
19.01–24.00	11,800 (61.9%)
24.01–28.00	2514 (13.2%)
>28.01	969 (5.1%)
Bath frequency (*n*, %)	
≤1 time/week	2525 (13.2%)
2–4 times/week	6900 (36.2%)
5–7 times/week	8670 (45.5%)
≥8 times/week	969 (5.1%)
Average humidity over past 6 years (%)	59.2 ± 13.2
Average temperature over past 6 years (°C)	28.7 ± 0.5

**Table 2:** Distribution of air pollution exposure for case and control populations.

Population		O_3_ (µg/kg·Day)	CO (mg/kg·Day)	SO_2_ (µg/kg·Day)	NO_2_ (µg/kg·Day)	PM2.5 (µg/kg·Day)	PM10 (µg/kg·Day)
Total	Mean ± SD	11.097 ± 1.624	0.150 ± 0.067	3.138 ± 1.980	4.212 ± 2.550	8.295 ± 3.503	12.895 ± 6.050
	Min	5.219	0.019	0.043	0.038	0.922	1.019
	25th	10.044	0.105	1.983	2.253	5.553	8.749
	50th	11.080	0.146	2.857	3.961	8.126	12.197
	75th	12.103	0.187	3.840	5.931	10.756	16.377
	Max	21.667	0.574	17.875	14.561	23.879	40.596
Atopic dermatitis	Case	10.810 ± 1.636	0.155 ± 0.059	3.246 ± 1.636	4.706 ± 2.492	8.644 ± 3.317	13.241 ± 5.469
	Control	11.105 ± 1.623	0.149 ± 0.067	3.135 ± 1.988	4.199 ± 2.550	8.286 ± 3.508	12.886 ± 6.064
Chronic hand eczema	Case	10.998 ± 1.502	0.160 ± 0.064	3.355 ± 1.990	4.558 ± 2.464	8.827 ± 3.308	13.565 ± 5.656
	Control	11.100 ± 1.627	0.149 ± 0.067	3.131 ± 1.980	4.202 ± 2.552	8.279 ± 3.508	12.875 ± 6.061
Urticaria	Case	10.914 ± 1.566	0.160 ± 0.063	3.332 ± 1.832	4.613 ± 2.484	8.852 ± 3.349	13.647 ± 5.703
	Control	11.111 ± 1.627	0.149 ± 0.067	3.123 ± 1.990	4.182 ± 2.553	8.253 ± 3.511	12.839 ± 6.072

**Table 3:** Association of second-hand smoke with inflammatory skin diseases.

	Atopic Dermatitis			Chronic Hand Eczema			Urticaria		
	Case (*n* = 481)	Control (*n* = 18,583)	*p*	Case (*n* = 560)	Control (*n* = 18,504)	*p*	Case (*n* = 1332)	Control (*n* = 17,732)	*p*
Never	362 (75.3%)	14,738 (79.3%)	**0.042**	393 (70.2%)	14,707 (79.5%)	**<0.001**	1010 (75.8%)	14,090 (79.5%)	**0.002**
<1 day/week	84 (17.5%)	2513 (13.5%)		109 (19.5%)	2488 (13.4%)		223 (16.7%)	2374 (13.4%)	
≥1 day/week	35 (7.3%)	1332 (7.2%)		58 (10.4%)	1309 (7.1%)		99 (7.4%)	1268 (7.2%)	

**Table 4:** Interactions between second-hand smoke (SHS) and air pollution exposure (OR (95% CI)).

		Atopic Dermatitis	Chronic Hand Eczema	Urticaria
O_3_	Q1	Ref.	Ref.	Ref.
	Q2	1.23 (0.71–2.14)	1.06 (0.64–1.75)	1.06 (0.75–1.50)
	Q3	0.88 (0.48–1.60)	0.82 (0.49–1.37)	0.99 (0.68–1.43)
	Q4	0.55 (0.27–1.14)	0.81 (0.45–1.44)	0.96 (0.66–1.40)
CO	Q1	Ref.	Ref.	Ref.
	Q2	1.87 (0.83–4.19)	0.97 (0.53–1.78)	0.98 (0.65–1.49)
	Q3	2.57 (1.16–5.69) *	1.16 (0.63–2.12)	0.84 (0.56–1.25)
	Q4	1.45 (0.64–3.29)	1.00 (0.55–1.82)	0.68 (0.45–1.03)
NO_2_	Q1	Ref.	Ref.	Ref.
	Q2	1.67 (0.72–3.88)	0.69 (0.38–1.26)	0.73 (0.48–1.09)
	Q3	2.38 (1.07–5.27) *	0.82 (0.47–1.43)	0.67 (0.45–1.00)
	Q4	2.07 (0.94–4.57)	1.00 (0.58–1.74)	0.65 (0.44–0.97) *
SO_2_	Q1	Ref.	Ref.	Ref.
	Q2	2.09 (0.99–4.36)	0.99 (0.56–1.77)	0.86 (0.57–1.29)
	Q3	1.75 (0.85–3.61)	0.91 (0.51–1.63)	0.97 (0.65–1.44)
	Q4	1.37 (0.64–2.92)	1.23 (0.68–2.21)	0.66 (0.43–1.01)
PM_2.5_	Q1	Ref.	Ref.	Ref.
	Q2	1.53 (0.79–2.97)	2.25 (1.22–4.15) **	1.04 (0.69–1.57)
	Q3	1.24 (0.63–2.41)	1.30 (0.70–2.41)	0.90 (0.61–1.33)
	Q4	1.01 (0.51–2.03)	1.61 (0.87–2.97)	0.93 (0.62–1.39)
PM_10_	Q1	Ref.	Ref.	Ref.
	Q2	1.77 (0.89–3.53)	1.80 (1.00–3.24)	1.02 (0.68–1.53)
	Q3	1.55 (0.78–3.09)	1.10 (0.60–2.01)	0.98 (0.62–1.44)
	Q4	1.12 (0.54–2.33)	1.50 (0.82–2.74)	0.76 (0.50–1.14)

Q1–Q4: first, second, third, and fourth quartiles of pollutant exposure level. * 0.01 < *p* < 0.05; ** 0.001 < *p* < 0.01.

## Data Availability

Data are available on request from the authors.

## Supplementary Materials

The following supporting information can be downloaded at https://www.mdpi.com/article/10.3390/ijerph191610011/s1: Figure S1. Geographical distribution of 20,138 students; Figure S2. Linear association between atopic dermatitis prevalence and adjusted daily dose of ambient air pollutants and environmental factors; Figure S3. Linear association between chronic hand eczema prevalence and adjusted daily dose of ambient air pollutants and environmental factors; Figure S4. Linear association between urticaria prevalence and adjusted daily dose of ambient air pollutants and environmental factors; Figure S5. Subgroup analysis of association between O_3_, SO_2_, PM_10_, and inflammatory skin diseases; Table S1. Questionnaire content relevant to the study; Table S2. Covariates between case and control populations for inflammatory skin diseases.
