# Supplementary material for: Synergistic Effects between Ambient Air Pollution and Second-Hand Smoke on Inflammatory Skin Diseases in Chinese Adolescents
Source: Int J Environ Res Public Health. 2022 Aug 13;19(16):10011. doi: 10.3390/ijerph191610011 (PMC9408277; doi:10.3390/ijerph191610011)
Supplement: Supplementary file 1 [file ijerph-19-10011-s001.zip › ijerph-1790322-supplementary.pdf]

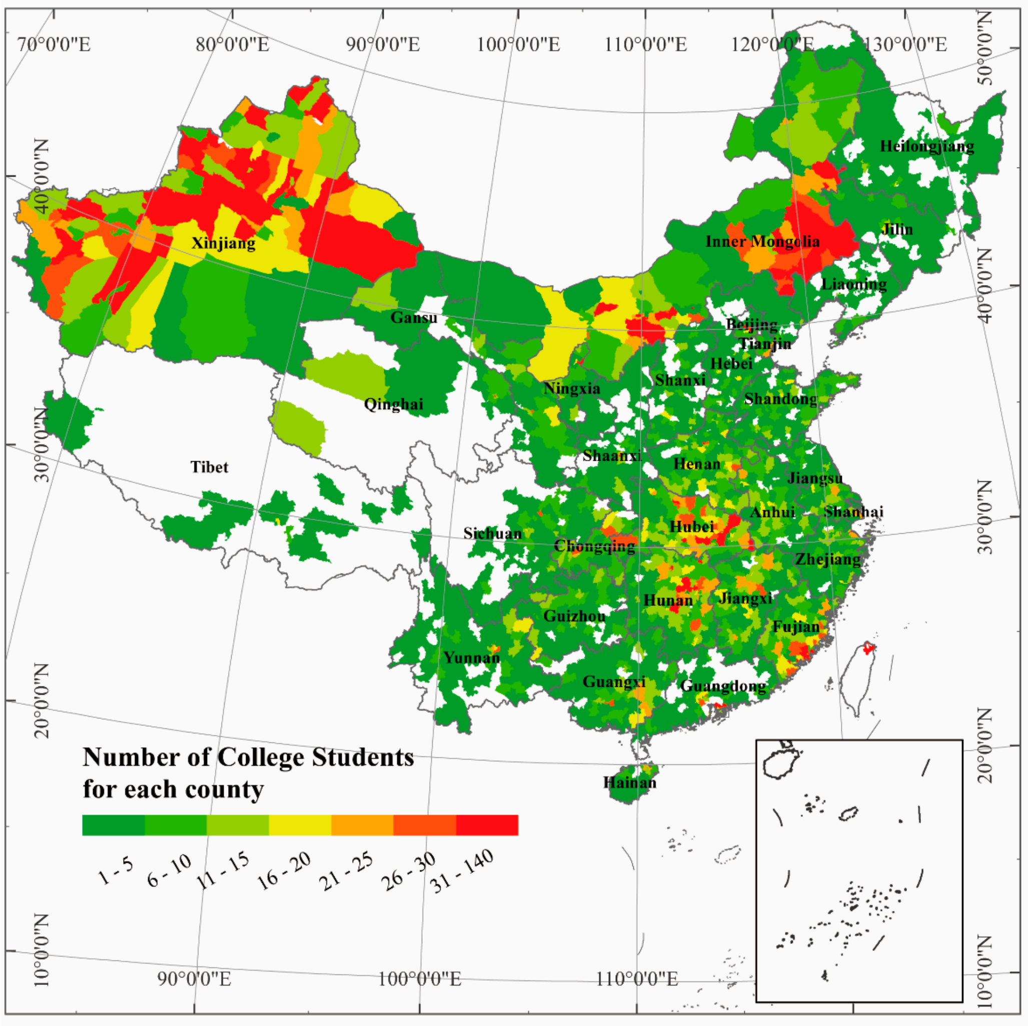

Supplementary Figure S1. Geographical distribution of 20,138 students.

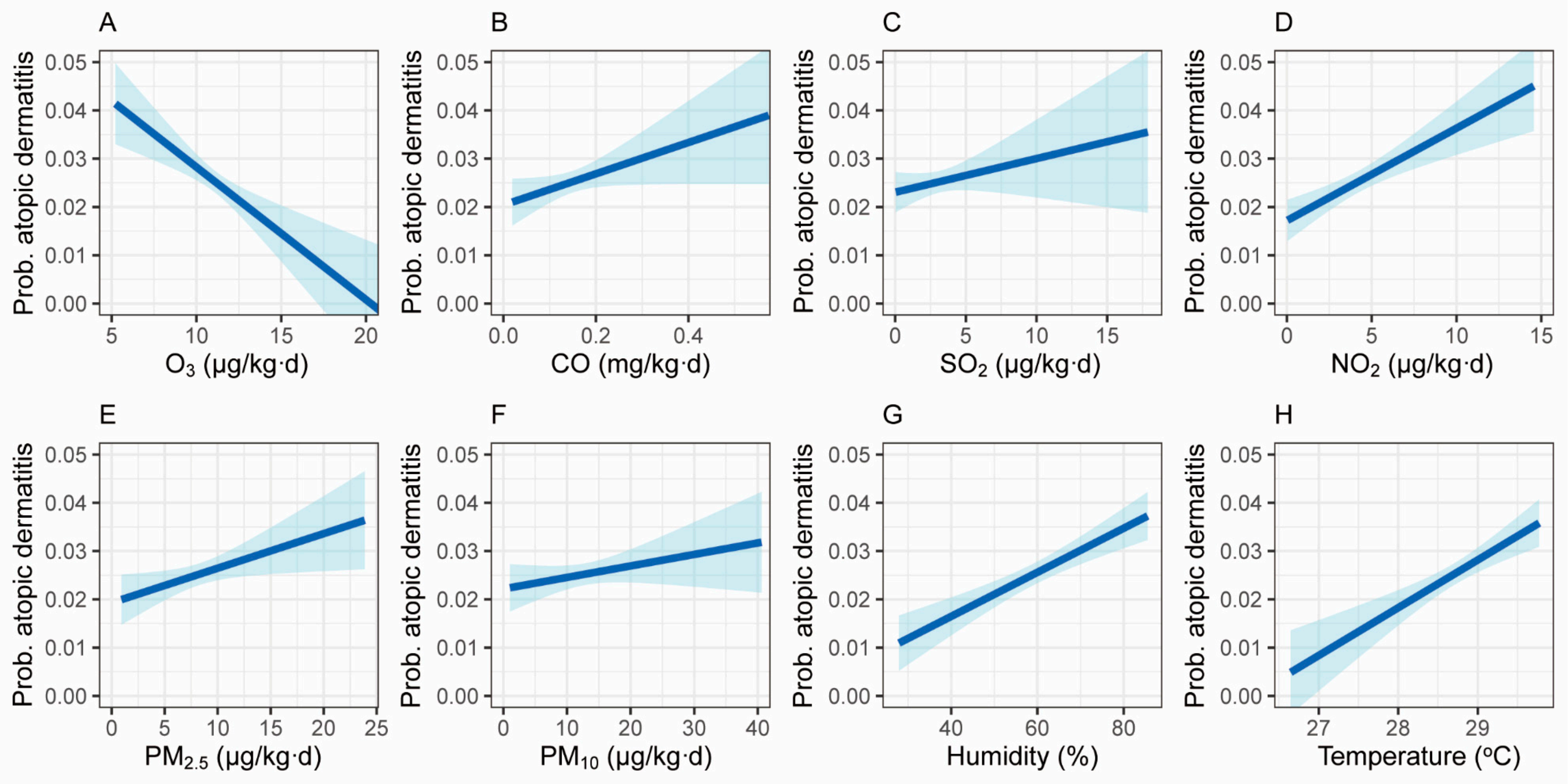

Supplementary Figure S2. Linear association between atopic dermatitis prevalence and adjusted daily dose of ambient air pollutants and environmental factors. Association between atopic dermatitis and O_3 (A), CO (B), SO_2 (C), NO_2 (D), $PM_{2.5}$ (E), PM_{10} (F), humidity (G) and temperature (H).

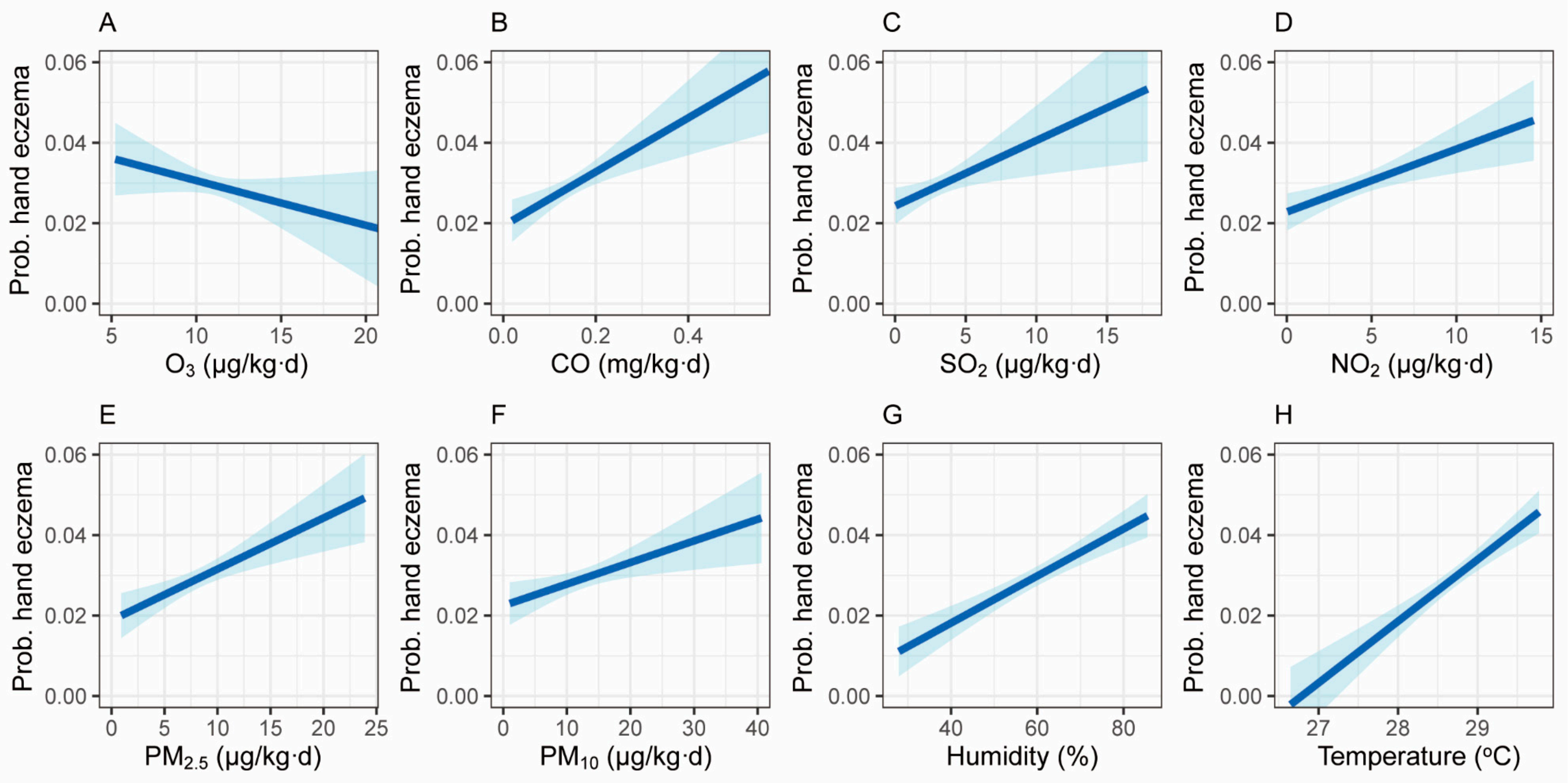

Supplementary Figure S3. Linear association between chronic hand eczema prevalence and adjusted daily dose of ambient air pollutants and environmental factors. Association between chronic hand eczema and O_3 (A), CO (B), SO_2 (C), NO_2 (D), $PM_{2.5}$ (E), PM_{10} (F), humidity (G) and temperature (H).

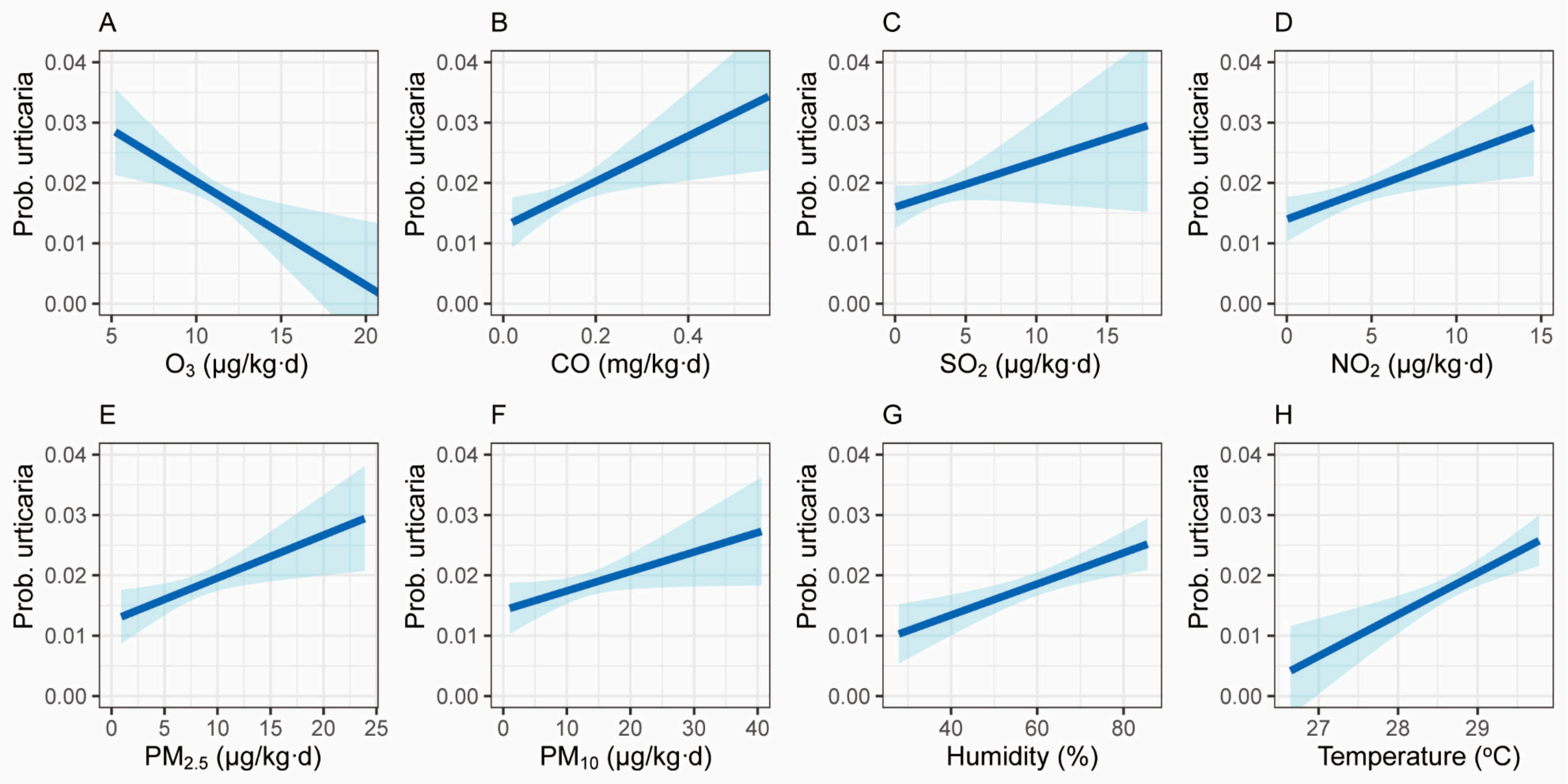

Supplementary Figure S4. Linear association between urticaria prevalence and adjusted daily dose of ambient air pollutants and environmental factors. Association between urticaria and O_3 (A), CO (B), SO_2 (C), NO_2 (D), $PM_{2.5}$ (E), PM_{10} (F), humidity (G) and temperature (H).

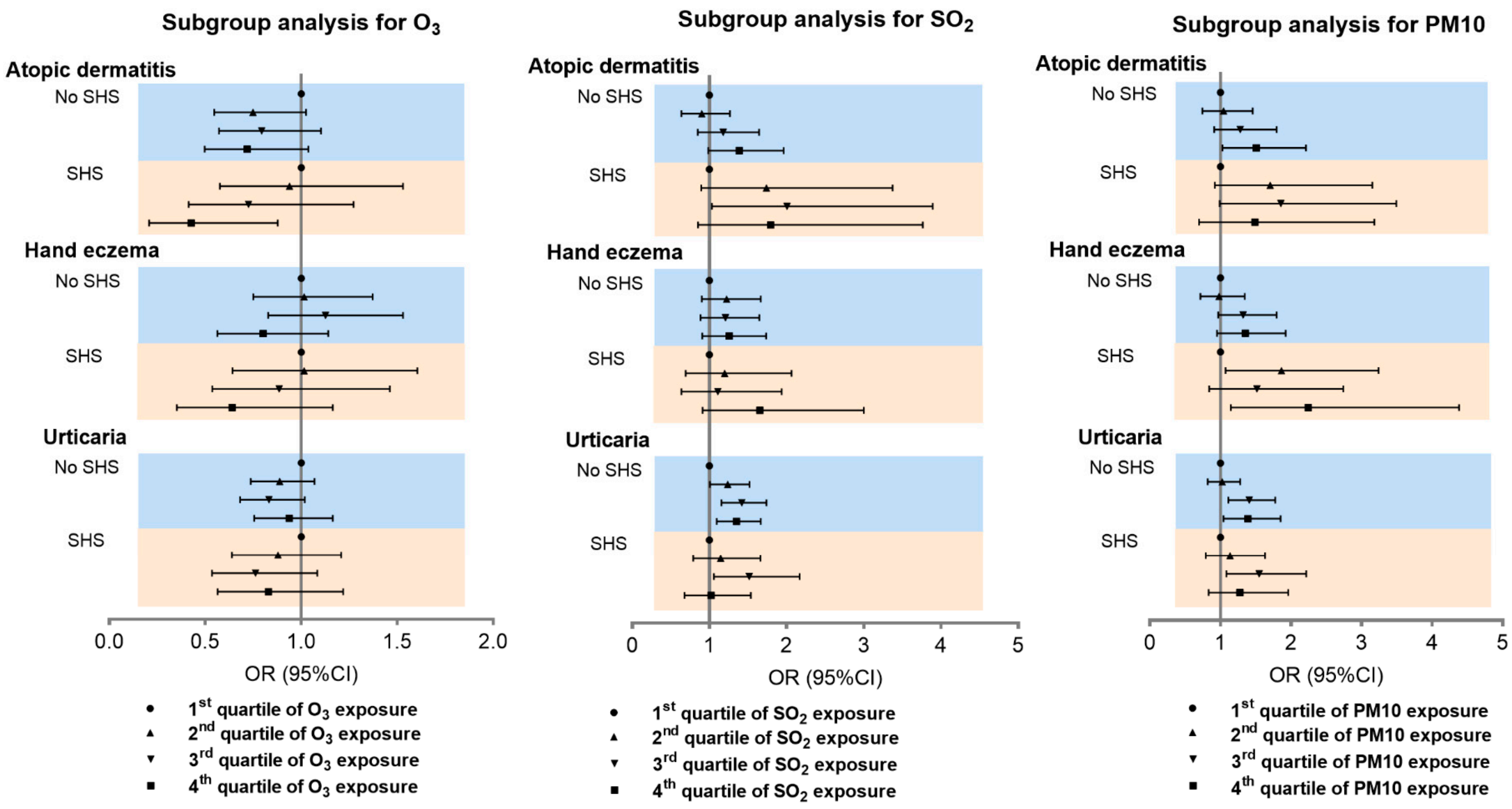

Supplementary Figure S5. Subgroup analysis of association between O_3, SO_2, PM_{10}, and inflammatory skin diseases.

Supplementary Table S1. Questionnaire content relevant to the study.

**How frequent do you usually take a shower in spring and autumn?**<br>A. ≤1 time/week B. 2 - 4 times/week C. 5 - 7 times/week<br>D. 8 - 10 times/week E. > 10 times/week
**How much is the annual income for your family?**<br>A. Less than 10,000 RMB B. 10,000 - 29,999 RMB C. 30,000 - 49,999 RMB<br>D. 50,000 - 99,999 RMB E. 100,000 - 199,999 RMB F. More than 200,000 RMB
**How often were you exposed to passive smoking (passive inhalation of smoke caused by other smokers in living or working environment) in the last month?**<br>A. Never B. < 1 day/week C. 1-2 days/week<br>D. 3-5 days/week E. 6-7 days/week
**What kind(s) of physical activity did you take part in over the last year? (Check all that apply)**<br>A. I hardly ever took exercise, with the frequency less than twice per month<br>B. Walking C. Jogging D. Bicycling (including spinning bike)<br>E. Ball games (football, basketball, badminton, etc) F. Dancing<br>G. Swimming H. Fitness (Strength training) I. Others

**How long have you been taking exercise?**<br>A. Never B. < 1 year C. 1 - 3 years D. 4 - 5 years E. > 5 years
**How often do you take exercise every week?**<br>A. < 1 hour B. 1 - 2 hours C. 3 - 6 hours D. ≥7 hours
**The actual duration of your sleep is usually ____ hours (fill in an integer)**

Supplementary Table S2. Covariates between case and control populations for inflammatory skin diseases.

Characteristic	Atopic dermatitis			Chronic hand eczema			Urticaria		
	Case (n = 481)	Control (n = 18583)	*p*	Case (n = 560)	Control (n = 18504)	*p*	Case (n = 1332)	Control (n = 17732)	*p*
Sex (n)									
male	246 (51.1%)	9567 (51.5%)	0.883	281 (50.2%)	9532 (51.5%)	0.534	675 (50.7%)	9138 (51.5%)	0.546
female	235 (48.9%)	9016 (48.5%)		279 (49.8%)	8972 (48.5%)		657 (49.3%)	8594 (48.5%)	
Family annual income (yuan, n)									
<10,000	32 (6.7%)	2014 (10.8%)	**0.001**	62 (11.1%)	1984 (10.7%)	0.413	94 (7.1%)	1952 (11.0%)	**<0.001**
10,000-29,999	90 (18.7%)	4061 (21.9%)		120 (21.4%)	4031 (21.8%)		216 (16.2%)	3935 (22.2%)	
30,000-49,999	88 (18.3%)	3228 (17.4%)		93 (16.6%)	3223 (17.4%)		264 (19.8%)	3052 (17.2%)	
50,000-99,999	109 (22.7%)	4102 (22.1%)		136 (24.3%)	4075 (22.0%)		317 (23.8%)	3894 (22.0%)	
100,000-199,999	105 (21.8%)	3720 (20.0%)		97 (17.3%)	3728 (20.1%)		317 (23.8%)	3508 (19.8%)	
≥200,000	57 (11.9%)	1458 (7.8%)		52 (9.3%)	1463 (7.9%)		124 (9.3%)	1391 (7.8%)	
BMI (n)									
<19.00	90 (18.7%)	3691 (19.9%)	0.887	121 (21.6%)	3660 (19.8%)	**0.020**	249 (18.7%)	3532 (19.9%)	0.501
19.01-24.00	296 (61.5%)	11504 (61.9%)		367 (65.5%)	11433 (61.8%)		843 (63.3%)	10957 (61.8%)	
24.01-28.00	70 (14.6%)	2444 (13.2%)		54 (9.6%)	2460 (13.3%)		182 (13.7%)	2332 (13.2%)	
>28.01	25 (5.2%)	944 (5.1%)		18 (3.2%)	951 (5.1%)		58 (4.4%)	911 (5.1%)	
Bath frequency (n)									

<=1 time/week	50 (10.4%)	2475 (13.3%)	**0.017**	54 (9.6%)	2471 (13.4%)	**0.009**	113 (8.5%)	2412 (13.6%)	**<0.001**
2-4 times/week	154 (32.0%)	6746 (36.3%)		187 (33.4%)	6713 (36.3%)		413 (31.0%)	6487 (36.6%)	
5-7 times/week	249 (51.8%)	8421 (45.3%)		287 (51.3%)	8383 (45.3%)		729 (54.7%)	7941 (44.8%)	
>=8 times/week	28 (5.8%)	941 (5.1%)		32 (5.7%)	937 (5.1%)		77 (5.8%)	892 (5.0%)	
